# Fibroblast phenotypes in different lung diseases

**DOI:** 10.1186/s13019-014-0147-z

**Published:** 2014-09-05

**Authors:** Heng Du, Dali Chen, Yubin Zhou, Zhaojie Han, Guowei Che

**Affiliations:** Department of Thoracic Surgery, West-China Hospital, Sichuan University, Chengdu, 610041 China

**Keywords:** Cancer associated fibroblasts, Phenotypes, Lung disease, Immunohistochemistry

## Abstract

**Background:**

The “seed and soil” hypothesis emphasizes the importance of interactions between tumor cells and their microenvironment. CAFs (Cancer associated fibroblasts) are important components of the tumor microenvironment. They were widely involved in cancer cells growth and metastasis. Fibroblasts may also play a role in inflammatory disease. The phenotype conversion of fibroblasts in lung diseases has not been investigated previously. We hypothesized that fibroblasts phenotypes may vary among different types of lung disease.

**Methods:**

The study included six types of lung tissues, ranging from normal lung to lung adenocarcinoma with lymphatic metastasis. Para-carcinoma tissues which were 2-cm-away from the tumor focus were also included in the analysis. The expression of target proteins including alpha-SMA (smooth muscle actin), FAP (fibroblast activation protein), vimentin, E-cadherin, and CK-19 (cytokeratin-19) were examined by immunohistochemistry. TGF-beta(transforming growth factor) and Twist were detected simultaneously in all samples.

**Results:**

A progressive increase in the levels of alpha-SMA, vimentin and CK-19 was observed in correlation to the degree of malignancy from normal lung tissue to lung adenocarcinoma with lymphatic metastasis, whereas E-cadherin expression showed the opposite trend. TGF-beta and Twist were detected in cancer tissues and inflammatory pseudotumors. None of the proteins were detected in para-carcinoma tissues.

**Conclusions:**

Fibroblast phenotypes varied according to the type and degree of lung malignancy and fibroblasts phenotypic conversion occurs as a gradual process with specific spatiotemporal characteristics. Similar fibroblast phenotypes in inflammatory diseases and cancer tissues suggested a correlation between inflammation and cancer and implied a common mechanism underlying the formation of fibroblasts in inflammatory diseases and lung cancer.

**Electronic supplementary material:**

The online version of this article (doi:10.1186/s13019-014-0147-z) contains supplementary material, which is available to authorized users.

## Background

The high degree of malignancy and heterogeneity of cancer cells have led researchers to focus on the study of malignant cells present in the epithelial compartment [1]. The resistance of certain cancer cells to treatment remains a critical issue [2]. In recent years, researchers have discovered that tumor microenvironment also plays an important role in tumor development. The interaction of the tumor microenvironment with cancer cells make the matters more complicated [2]-[5]. Tumor microenvironment may play a key role in tumor invasion and can indirectly affect prognosis [2],[6]. The development of resistance against chemotherapeutic drugs brought attention to the fact that the soil (tumor microenvironment) is as important as seeds (cancer cells).

Specialized fibroblasts named cancer-associated fibroblasts (CAFs) are characterized by “cross-talk” with cancer cells and have become novel targets for cancer therapy [2],[6],[7]. CAFs can be formed through several ways. The way named EMT (epithelial-mesenchymal transition) was important. Studies have demonstrated that in human breast and prostate cancer, TGF-β and Twist secreted by tumor cells may induce EMT and promote the formation of CAFs [3],[7]-[9]. CAFs express high levels of α-SMA (smooth muscle actin) and vimentin [3],[10],[11], whereas they are negative for cytokeratin and E-cadherin [3],[4],[12]. Normal fibroblasts suppress tumor genesis by inhibiting the proliferation of the adjacent epithelium, CAFs play an opposite role [4].

Vicent S et al. [13] made a cross-species functional characterization of mouse and human lung CAFs and finally found that CAFs supported the growth of lung cancer cells in vivo by secretion of soluble factors that directly stimulate the growth of tumor cells. They also found that IL-6 (interleukin-6) secreted by CAFs promoted cancer cells growth. Besides, CXCL-12/CXCR4 axis which existed between the cross-talk of CAFs and non-small lung cancer cells was also contributed to the proliferation of cancer cells [14]. Some proteins expressed by CAFs such as matrix metalloproteinase (MMP)-2, α-SMA, podoplanin and carbonic anhydrase (CA) IX may correlated with the prognosis of lung cancer [15]-[17].

The phenotype conversion from normal fibroblasts to CAF was associated with carcinogenesis but whether the CAF phenotype varies among cancer tissues remain to be elucidated. We hypothesized that TGF-β and Twist participated in lung carcinogenesis. In previous studies, the role of CAFs was investigated by assessing their tissue-specific expression only in one type of human cancer such as human prostate cancer or human breast cancer [18],[19]. We analyzed fibroblasts in a set of samples which including chronic inflammation, normal lung tissue, atypical adenomatous hyperplasia, carcinoma in situ, lung adenocarcinoma, and lung adenocarcinoma with lymphatic metastasis. Para-carcinoma tissues were collected from the relevant patients. A comparative analysis of these tissues was performed to examine the characteristics of the phenotypic conversion of fibroblasts.

## Methods

### Patients

Patient samples were obtained from the department of thoracic surgery of West China Hospital. The study was approved by the West China Hospital Ethics Committee (year 2013, No.32). All patients signed informed consent forms. The distribution of patients was as follows: N (pulmonary bulla) (n = 20), I (inflammatory pseudotumor) (n = 22), H (atypical adenomatous hyperplasia)(n = 19), CIS (carcinoma in situ) (n = 13) and CISJ (corresponding adjacent tissues), A (lung adenocarcinoma without lymph node metastasis) (n = 26) and AJ (corresponding adjacent tissues), and AM (lung adenocarcinoma with lymphatic metastasis) (n = 27) and AMJ (corresponding adjacent tissues). None of the patients had received chemotherapy or radiotherapy before surgery. Tissues from patients with pulmonary bulla who were treated surgically were used as controls because of the lack of available healthy donor tissues. Besides, the CIS samples were not as much as other samples for the reason that cancer was very difficult to diagnose in an early stage. Nearly all the CIS samples came from patients whose tumors were found by regular medical examination. Unlike other pathological stages of lung cancer, samples of CIS (carcinoma in situ) of lung cancer is relatively hard to found and the size of samples was very small. We could not obtained enough sections from a single patient. Histological analyses of tissue samples were performed independently by two pathologists who were blinded to patients’ clinical information according to World Health Organization criteria.

### Methods

#### Immunohistochemistry

4.5 μm thick serial sections of formalin-fixed and paraffin-embedded tissue samples were heated in citrate buffer (0.01 M) for 30 min. Endogenous peroxidase activity was quenched by incubation in 0.5% H_2_O_2_. Sections were then blocked with serum and incubated with the respective primary antibodies at 4°C overnight. Primary antibodies and their dilutions were as follows: mouse monoclonal anti α-SMA (ZXGB BIO, Beijing, CHINA, 1:100); rabbit polyclonal anti FAP (Assay Biotech, U.S.A. 1:200); mouse monoclonal anti vimentin, mouse monoclonal anti-E-cadherin, mouse monoclonal anti- CK-19, and rabbit polyclonal anti TGF-β (all obtained from ZXGB BIO, Beijing, CHINA, and used at 1:100); and mouse monoclonal anti-Twist (Santa Cruz Biotechnology, Dallas, U.S.A. 1:100). Negative control sections were concurrently obtained from each patient and incubated in PBS (0.01 M). Sections were then incubated in the corresponding secondary antibody (anti-mouse IgG and anti-rabbit IgG, both from ZSGB BIO, Beijing, CHINA,1:200), then in the avidin biotin complex reagent (ZSGB BIO, Beijing, CHINA,1:100) for 1 h at room temperature. Next, sections were visualized by using 3, 3- diaminobenzidine (25 μL 3% H_2_O_2_ and 5 mL DAB) for 4 min and counterstained with nuclear fast red for 2 min.

#### Immunohistochemistry analysis

For the markers expressed in cancer cells, high magnification (40 × objective) was used to count the individual cancer cells and a total of 200 cancer cells were scored for each high-power field. For every section, five fields were selected randomly. Normal alveolar epithelial cells stained red by nuclear fast red were selected as negative controls. According to Friedrichs et al. [18], a staining index (SI, values 0, 1, 2, 3, 4, 6, and 9) was calculated as a product of staining intensity (0–3) and the proportion of positive cells (0% = 0, 1 – 10% = 1, 11 – 50% = 2, >50% = 3) [18]. For CAFs, the SI was calculated as a product of staining intensity (0–3) and the staining extent (0% = 0, 1 –10% = 1, 11 – 50% = 2, >50% = 3).

#### Statistical analysis

The Wilcoxon rank sum test was used to compare the marker status among the six disease types. Correlation analyses between TGF-β and α-SMA, TGF-β and E-cadherin, TGF-β and vimentin, Twist and α-SMA, Twist and vimentin, and Twist and E-cadherin were performed by comparing the SI (staining index) for each protein in the same disease using Spearman’s rank test. A P value (two sides) < 0.05 was considered to indicate statistical significance.

## Results

### Clinical findings

The study included 127 patients (70 men and 57 women) with a mean age of 55 years (range: 18–74 years). The presence of lymph node metastasis was confirmed by intraoperative frozen section analysis and none of the patients had distant metastasis.

### Fibroblasts phenotypic conversion characteristics

#### Detection of fibroblasts and epithelial markers

**Figure 1:**
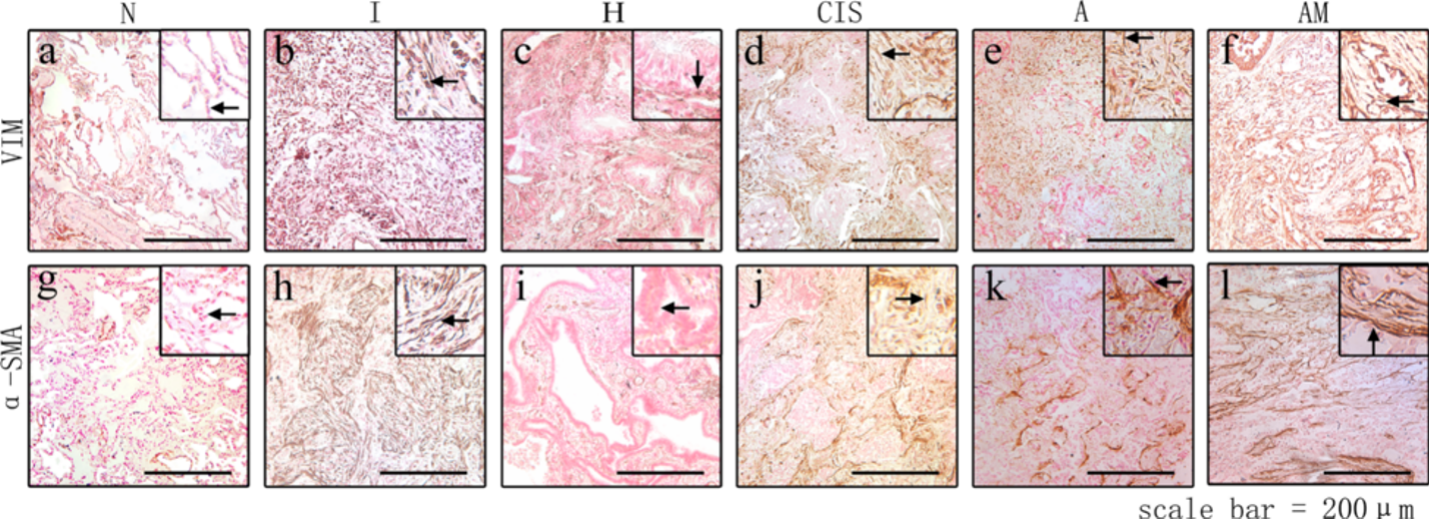
**Expression of alpha-smooth muscle actin and vimentin in lung tissues from N to AM.** Fibroblast markers [alpha-smooth muscle actin (α-SMA) **(a-f)**, vimentin **(g-l)**] were detected by immunohistochemistry. As shown in figure, both α-SMA and vimentin were stained positively in the cytoplasm of CAFs. Magnification: 40×.

α-SMA was detected in benign and malignant tissues and its expression was localized to blood vessels and airway ducts in N and H. Stromal cells were positive for α-SMA in inflammatory pseudotumor as well as in all three pathological stages of adenocarcinoma, although with different staining intensity. α-SMA expression was lower in CIS (SI ranged from 1 to 4 and the median was 2) than in A (SI : 1 to 6, median was 4) (P = 0.026), whereas AM (SI: 4 to 9, median was 6) showed higher expression than A (P = 0.009). Vimentin expression was negative in N and H but positive in I. In the cancer samples, Vimentin expression was higher in A (SI: 2 to 6, median was 3.5) than in CIS (SI: 0 to 4, median was 2) (P = 0.017) and it was higher in AM (SI: 3 to 9, median was 6) than in A (P = 0.022). In general, α-SMA and vimentin expression increased gradually from N to AM (Figure 1).

**Figure 2:**
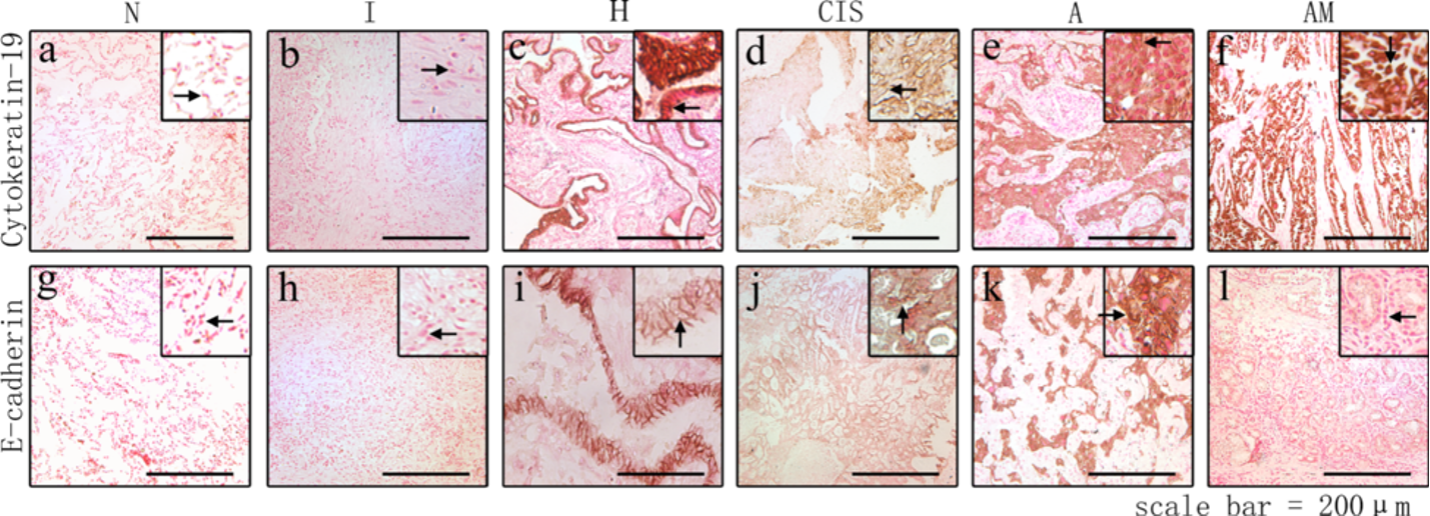
**Expression of E-cadherin and cytokeratin-19 in lung tissues from N to AM.** Epithelial markers [E-cadherin **(a-f)** and cytokeratin-19 (CK-19) **(g-l)**] were detected by immunohistochemistry. E-cadherin was located in cell membrane while CK-19 was located in cytoplasm. Magnification: 40×.

The epithelial marker E-cadherin was expressed at high levels in the glandular epithelium of H. E-cadherin staining intensity was lower in most CIS (SI: 0 to 3, median was 2) samples than in H (SI: 2 to 4, median was 3) samples (P = 0.008), and it was higher in A (SI: 0 to 4, median was 3) than in CIS (P = 0.827). E-cadherin expression was significantly lower in AM (SI: 0 to 1, median was 1) than in A (P < 0.001). CK-19 showed strong staining in H and was expressed at higher levels in A than in CIS (P = 0.003) and in AM (SI: 4–9 median was 6) than in A (P = 0.035). Cancer epithelial cells expressed more CK-19 in the samples from CIS to AM. The expression of epithelial markers was negative in N and in I (Figure 2).

#### Immunohistochemical detection of TGF-β, FAP, and Twist

**Figure 3:**
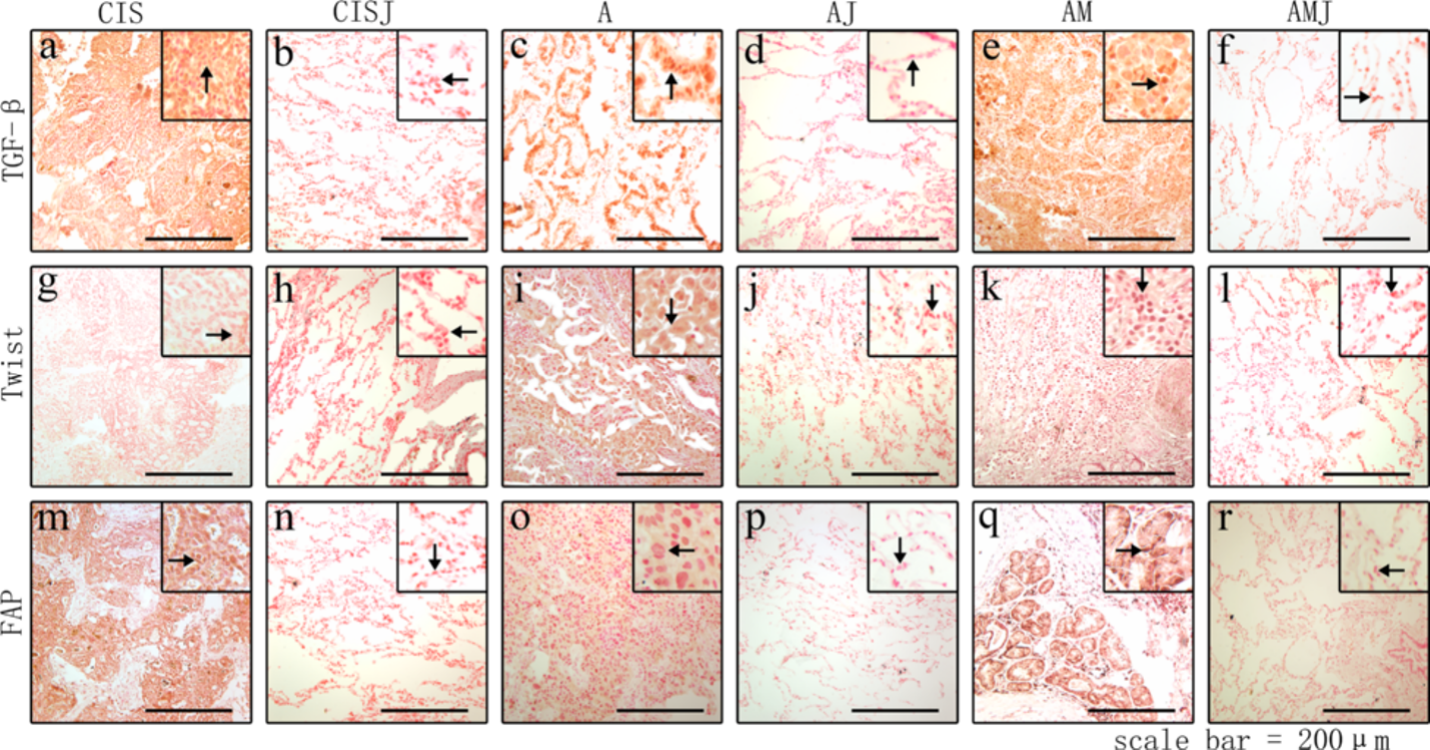
**Expression of TGF-β, FAP and Twist in cancer tissues and para-carcinoma tissues.** Key factors in the cell signaling pathway including TGF-β **(a-f)** and Twist **(g-l)** were detected by immunohistochemistry. The expression of TGF-β and Twist in I (inflammation pseudotumor) is not included in the figure. In these samples, TGF-β was located in cytoplasm **(a, c, e)** and Twist **(g, I, k)** can be detected both in cytoplasm and nuclei of cancer cells. FAP, a marker of CAFs, was expressed in the cytoplasm of cancer cells rather than in the stroma of cancer tissues **(m, o, q)**. This novel result may indicate that some cancer cell could secret FAP just like CAFs or these cancer cells secreting FAP may be a novel origin of CAFs. However, TGF-β **(b, d, f)**, Twist **(h, j, l)** or FAP **(n, p, r)** were negatively expressed by all of the para-carcinoma tissues. Magnification: 40×.

**Figure 4:**
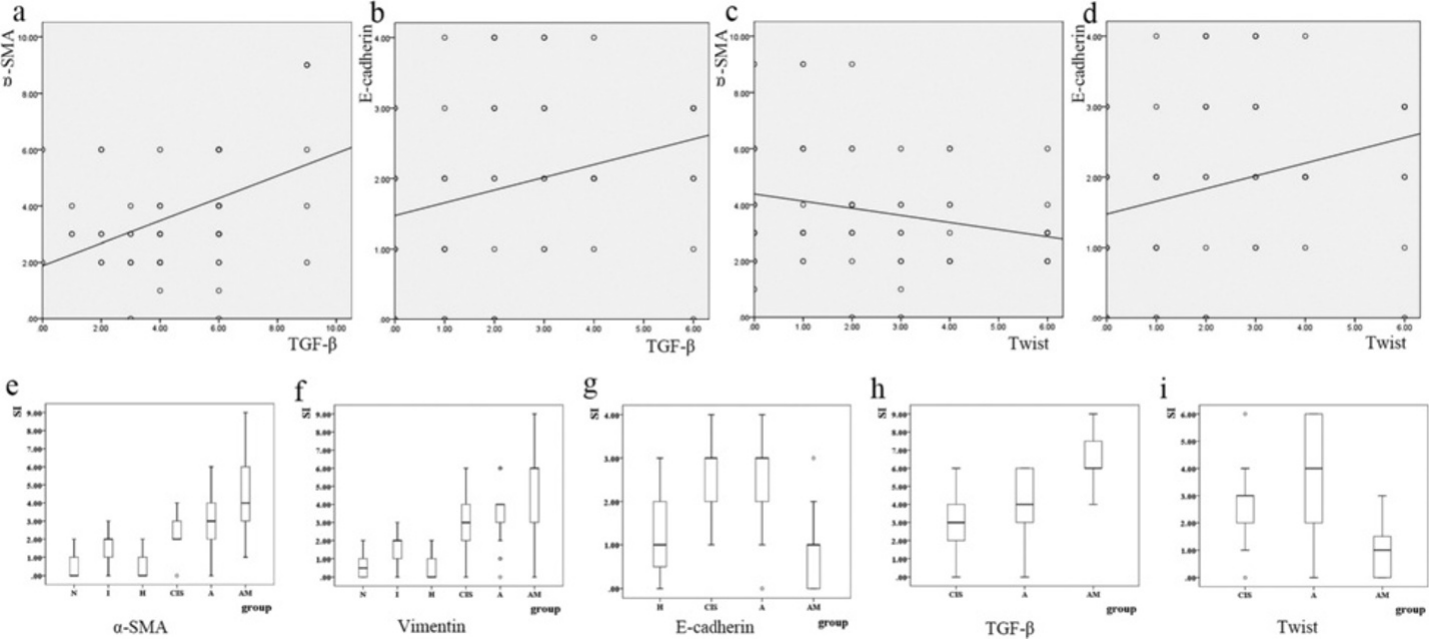
**Statistical analysis of the relevant factors.** The first row showed the correlation analysis between TGF-β and α-SMA **(a)**, TGF-β and E-cadherin **(b)**, Twist and α-SMA **(c)** and Twist and E-cadherin **(d)** were performed in all the 66 lung cancer samples (including CIS, A, AM). Each cycle in the figure represented one lung cancer sample. But some cycles were overlapped because these samples had a same score. α-SMA expression was lower in CIS than in A (P = 0.026), whereas AM showed higher expression than A (P = 0.009) (Figure 4 **e**). Vimentin expression was negative in N and H but positive in I. In the cancer samples, Vimentin expression was higher in A than in CIS (P = 0.017) and it was higher in AM than in A (P = 0.022) (Figure 4 **f**). E-cadherin staining intensity was lower in most CIS than in H (P = 0.008), and it was higher in A than in CIS. E-cadherin expression was significantly lower in AM than in A (P < 0.001) (Figure 4 **g**). TGF-β expression followed a similar pattern than that of α-SMA and vimentin, with positive expression in I, CIS, A, and AM. TGF-β was expressed at higher levels in AM than in A (P < 0.001). No statistical significance was seen between CIS and A (P = 0.392) (Figure 4 **h**). Twist expression was seen in I, A and AM (Figure 4 **i**).

The expression of TGF-β, FAP, and Twist was analyzed by IHC. Unlike the four markers which were detected in interstitial cells (such as α-SMA and vimentin) or the cancer cells membrane (such as CK-19), TGF-β and FAP expression was detected in the cytoplasm of cancer cells while twist was located in both cytoplasm and nucleus. The three factors were expressed in I, CIS, A, and AM, whereas N, H, but para-carcinoma tissues were negatively stained (the results of N, I, and H were not included in Figure 3). TGF-β was expressed at higher levels in AM (SI: 3 to 9, median was 6) than in A (SI: 2–9 median was 3.5) (P < 0.001). No statistical significance was seen between CIS and A (P = 0.392). On the other hand, Twist expression was seen in A and AM. FAP expression was positive in the matrix of cancer cells and negative in that of CAFs. FAP expression was detected in cancer cells of CIS, A and AM, although the differences in expression levels among CIS, A, and AM did not reach statistical significance (Figures 3 and 4).

#### Correlation analysis

All the lung cancer samples (including CIS, A and AM, totally 66 samples) expressed different levels of α-SMA, vimentin, E-cadherin, Twist and TGF-β. We performed a correlation analysis between TGF-β and α-SMA, TGF-β and vimentin, TGF-β and E-cadherin, Twist and α-SMA, Twist and E-cadherin and Twist and α-SMA in all the cancer samples. A significant positive correlation was observed between TGF-β and α-SMA and between TGF-β and vimentin expression (Spearman’s rank correlation coefficient r = 0.396, p = 0.001; r = 0.404, p = 0.009, respectively). A significant inverse correlation was observed between TGF-β and E-cadherin (r = −0.449, p < 0.001). However, a significant positive correlation was observed between Twist and E-cadherin expression (r = 0.318, p = 0.009). The relationship between Twist and α-SMA did not reach statistical significance (p = 0.064) (Figure 4).

### Spatial characteristics of the phenotypic conversion of cancer-associated fibroblasts

The spatial pattern of the phenotypic conversion of fibroblasts was examined by detecting marker protein expression in para-carcinoma tissues using the same methods. The results showed that adjacent tissues stained negative for all the marker proteins analyzed (Figure 3).

## Discussion

In previous studies, co-implantation of tumor cells and CAFs into immunodeficient mice induced tumor growth at a faster rate than the implantation of cancer cells alone or that of normal fibroblasts [19],[20]. Cytokines secreted by CAFs may contribute to destroy the basement membrane to facilitate tumor invasion and metastasis [21].

In the present study, we used six kinds of lung disease to examine the spatiotemporal characteristics of the phenotypic conversion of fibroblasts. A mild, progressive increasing trend in CAFs was observed from CIS, A to AM tissues. This could be explained as follows: the expression of α-SMA and vimentin in the stroma were increased from N to AM and both factors followed a similar pattern of upregulation. The upregulation of α-SMA promotes tumor formation and metastasis [22]. However, α-SMA expression was also positive in stromal cells of I (inflammatory pseudotumor), similar to that in tumors (P = 0.059). Increased TGF-β up-regulated the expression of α-SMA in I and in lung cancer (r = 0.396, p = 0.001). Vimentin, whose up-regulation is an indicator of EMT, showed a similar staining pattern among the different disease stages. Vimentin overexpression has been detected in several types of tumors, including lung adenocarcinoma, and its expression is correlated with tumorigenesis, invasion, and metastasis [23]-[25]. In addition to the markers of CAFs, we analyzed epithelial markers, including E-cadherin and CK-19. The down-regulation of these two epithelial markers accompanied by the up-regulation of α-SMA and vimentin is considered a key process associated with EMT [26],[27]. E-cadherin may play a role in suppressing tumor metastasis. Liu et al. showed that the impaired integrity of the E-cadherin-catenin system is responsible for the reduced or even negative expression of E-cadherin [28],[29]. In addition, Twist expression is associated with aberrant E-cadherin expression. CK-19 is frequently detected in serum as the fragment CYFRA −21 [30]-[32], and its expression in parenchyma cells is consistent with the results of the present study. The upregulation of CK-19 expression in cancer epithelial cells is associated with tumor invasion and lymphatic metastasis [33]-[35], which is also consistent with our results. However, the association between high CK-19 reactivity and cancer and its role in metastasis should be further investigated.

TGF-β, Twist and FAP were expressed at various levels in the different tumor tissues from CIS to AM. TGF-β is a multifunctional protein that induces fibrosis of lung tissue, tumor invasion, and metastasis by promoting EMT, and EMT takes an active part in fibrotic disease, as well as in cancer cell movement and invasion [36]-[39].

The involvement of TGF-β in EMT may require its association with Smad and Snail signaling or the activity of the Ras pathway; however, activated Smad or H-Ras may not be sufficient for the EMT process without the presence of TGF-β [40]. Ronnov-Jessen et al. suggested that TGF-β is the only factor required for the conversion of normal fibroblasts into CAFs [41]. Furthermore, Fibroblasts secrete TGF-β to remain active and promote their own proliferation [3]. The transcription factor Twist exerts a similar function to that of TGF-β [8],[9],[42],[43], and its upregulation is correlated with alterations in E-cadherin expression [44]. However, the present study showed the opposite results. A significant positive relationship was observed between Twist and E-cadherin expression (r = 0.318, p = 0.009). Comparison of Twist and E-cadherin expression between CIS and A showed a negative correlation. These results indicated a discordant pattern of expression of Twist, particularly in association with the process of tumor metastasis, which led us to hypothesize that Twist expression may be an indicator of metastasis in lung adenocarcinoma.

Among the six types of tissues, I (inflammatory pseudotumor) exhibited an abnormal pattern of immunostaining and was positive for α-SMA, vimentin, as well as TGF-β (Figure 1). TGF-β is among the factors secreted during the inflammatory response [45] and may promote the upregulation of α-SMA in response to inflammation. In inflammatory diseases such as Hepatitis B and inflammatory bowel disease, malignant transformation may lead to the development of hepatocellular carcinoma and colorectal carcinoma, respectively. The results of the present study suggest that benign inflammatory diseases may progress and develop into malignant lung tumors.

In addition to α-SMA and vimentin, FAP, is also considered to be a specific marker for CAFs [46]. However, our results showed that FAP was expressed in the cytoplasm of cancer cells rather than in the cytoplasm of CAFs, and no statistically significant differences were detected among CIS, A, and AM tissues. TGF-β expression was also localized to the matrix of cancer cells, which was not consistent with the results reported by Xing et al. These two novel phenomena revealed by our findings indicated that cancer cells might have a similar function as CAFs and CAFs may not originate exclusively from normal epithelial cells but may also be derived from mutant epithelial cells, including cancer cells [2].

Both lung inflammatory pseudotumor and atypical adenomatous hyperplasia might have something to do with malignant lung diseases. Lung inflammatory pseudotumor, whose etiology was still unknown, was a relative rare benign lung tumor and account for about 0.7% of primary pulmonary and bronchial tumors. [47] This benign lung disease had malignant biological behavior including locally invasive, aggressiveness and unfavorable evolution which needed extensive pulmonary resection to prevent local recurrence. Though no detail data about the incidence and the probability that the patients with lung cancer resulting from inflammatory pseudotumor was provided, it was better to have this lesion resected as early as possible [47]. Atypical adenomatous hyperplasia may be the adenoma in an adenoma–carcinoma sequence in the lung periphery, leading to the development of adenocarcinoma. Atypical adenomatous hyperplasia had a low morbidity of 4.4% and 9.6% [48],[49]. The incidence was higher in patients bearing lung cancer especially in those with lung adenocarcinoma (15.6% vs 23.2%) [50]. It had been demonstrated that atypical adenomatous hyperplasia might result in lung squamous carcinoma. Whether or not it would result in lung adenocarcinoma still need more research [51].

The present study analyzed a series of lung diseases simultaneously. We used longitudinal and cross-sectional analyses to investigate phenotypic variations in fibroblasts. While previous studies compared cancer cells to normal tissues in a static manner, our research reflected the dynamic changing process of fibroblasts. In addition to their role in promoting tumor invasion and metastasis, their function in providing a microenvironment for tumor cells similar to that of soils for seeds, fibroblasts were shown to undergo phenotypic conversion in a specific spatiotemporal pattern. Considering the progressive emergence of drug resistant phenotypes in cancer, our results suggest that activated fibroblasts may represent a potential target for molecular targeted therapy in lung cancer and provide a correlation between inflammatory diseases and cancer. The limitations of the present study were mainly derived from the lack of sample. Samples of CIS were specially limited for it was very hard to diagnose cancer at very early stage. The differences between our results and those of previous studies could be related to the small number of samples included in the analysis. In addition, tumor tissues consisted of several types of cells, which could lead to the differential expression of the factors analyzed.

In the present study, we detected seven types of factors. TGF-β showed a positive role in EMT and facilitated the generation of CAFs. Because Twist showed a similar function, we were unable to determine whether one of the factors plays a key role or if both are critical for EMT. If both TGF-β and Twist are essential for EMT, assessment of the potential cross-talk between the two factors would be important. However, IHC is insufficient to examine this issue, which we plan to address using different methods in future studies.

## Conclusions

Fibroblast phenotypes vary according to the type and degree of lung diseases and their phenotypic conversion occurs as a gradual process with specific spatiotemporal characteristics. Similar fibroblast phenotypes in inflammatory diseases and cancer tissues suggested a correlation between these two disease types and a common mechanism underlying the formation of fibroblasts in inflammatory diseases and lung cancer. The present study want to provide a new idea about cancer therapy.

## Authors’ original submitted files for images

Below are the links to the authors’ original submitted files for images. Authors’ original file for figure 1 Authors’ original file for figure 2 Authors’ original file for figure 3 Authors’ original file for figure 4

## Abbreviations

A: Lung adenocarcinoma without lymph node metastasis
AJ: Lung tissue 2-cm away from the tumor
AM: Lung adenocarcinoma with lymph node metastasis
AMJ: Lung tissue 2-cm away from the tumor
CAFs: Cancer associated fibroblasts
CIS: Carcinoma in situ
CISJ: Lung tissue 2-cm away from the tumor
CK-19: Cytokeratin-19
FAP: Fibroblast activation protein
H: Atypical adenomatous hyperplasia
I: Inflammatory pseudotumor
N: Normal lung tissue
TGF-β: Transforming growth factor-β
α-SMA: α-smooth muscle actin
SI: Staining index
