# Supplementary figures and images for: Fibroblast phenotypes in different lung diseases
Source: J Cardiothorac Surg. 2014 Sep 5;9:147. doi: 10.1186/s13019-014-0147-z (PMC4173054; doi:10.1186/s13019-014-0147-z)

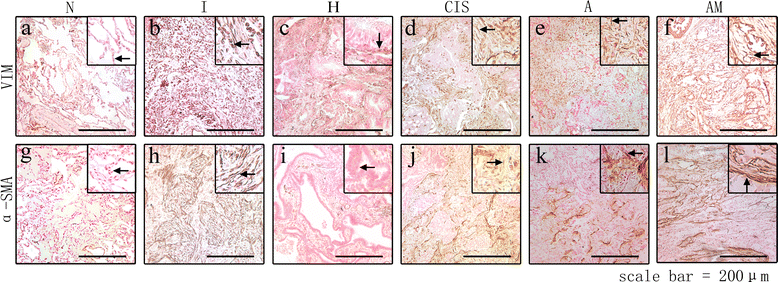

Supplement: Supplementary file 1 — Authors’ original file for figure 1 [file 13019_2014_147_MOESM1_ESM.gif]

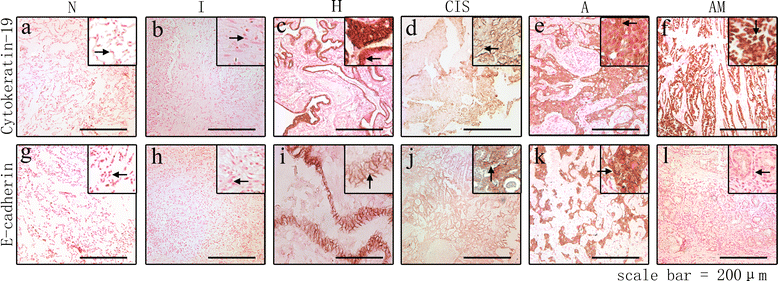

Supplement: Supplementary file 2 — Authors’ original file for figure 2 [file 13019_2014_147_MOESM2_ESM.gif]

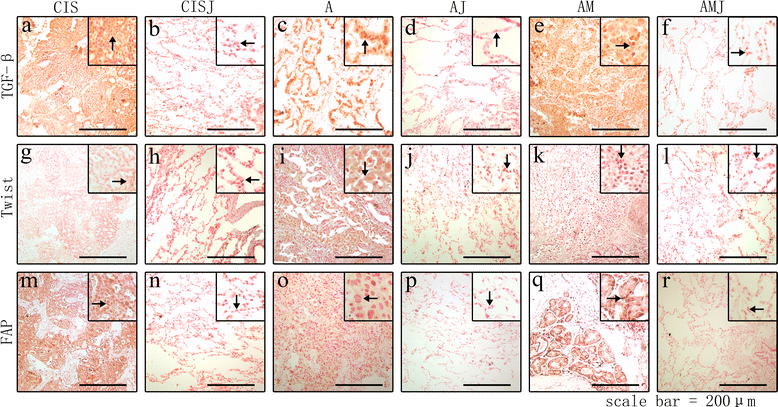

Supplement: Supplementary file 3 — Authors’ original file for figure 3 [file 13019_2014_147_MOESM3_ESM.gif]

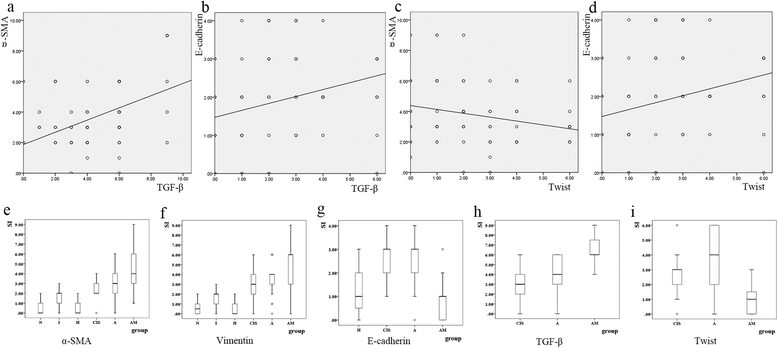

Supplement: Supplementary file 4 — Authors’ original file for figure 4 [file 13019_2014_147_MOESM4_ESM.gif]
